# PP03 Economic Evaluation Of Recombinant Thyroid-Stimulating Hormone Combined With Radioiodine Ablation For Patients With Differentiated Thyroid Cancer

**DOI:** 10.1017/S0266462325102237

**Published:** 2025-12-29

**Authors:** Mariana Fachi, Ludmila Gargano, Haliton Oliveira Junior, Rosa Camila Lucchetta, Layssa Oliveira

## Abstract

**Introduction:**

Differentiated thyroid cancer (DTC) treatment involves thyroidectomy and radioiodine ablation. Some patients have contraindications to induced endogenous hypothyroidism or cannot produce endogenous thyroid-stimulating hormone (TSH), preventing ablation unless recombinant TSH is used. This study aimed to evaluate the cost effectiveness of recombinant TSH followed by ablation in patients with DTC from the Brazilian Unified Health System perspective.

**Methods:**

An economic evaluation was conducted to estimate the incremental cost-effectiveness ratio of radioiodine ablation (with prior recombinant TSH), compared with the absence of these procedures in patients who, after thyroidectomy, are unable to induce endogenous hypothyroidism. A Markov model was built with three health states: no recurrence, recurrence, and death. The outcomes evaluated were quality-adjusted life years (QALYs) and life years (LYs) gained. Direct medical costs were included over a 30-year time horizon, with a five percent discount rate applied to both costs and outcomes. A deterministic and probabilistic sensitivity analysis was also performed.

**Results:**

In general, the combination of recombinant TSH with radioiodine ablation proved to be dominant compared with not performing ablation, resulting in lower costs (BRL25,062 versus BRL26,941 [USD10,271 versus USD11,041]) and better clinical outcomes (QALYs 9.69 versus 9.55 and LYs 10.82 versus 10.57). This result was mainly due to a statistically significant difference in recurrence rates. In the probabilistic sensitivity analysis, most simulations showed greater clinical benefit with recombinant TSH and ablation than no ablation. Additionally, the deterministic analysis indicated that the correction factor applied to costs and recurrence risk were the most impactful parameters in the economic model.

**Conclusions:**

The combination of recombinant TSH with radioiodine ablation was considered dominant in terms of QALYs and LYs gained, compared with no ablation, meaning the evaluated technology resulted in greater clinical benefit and lower cost.

